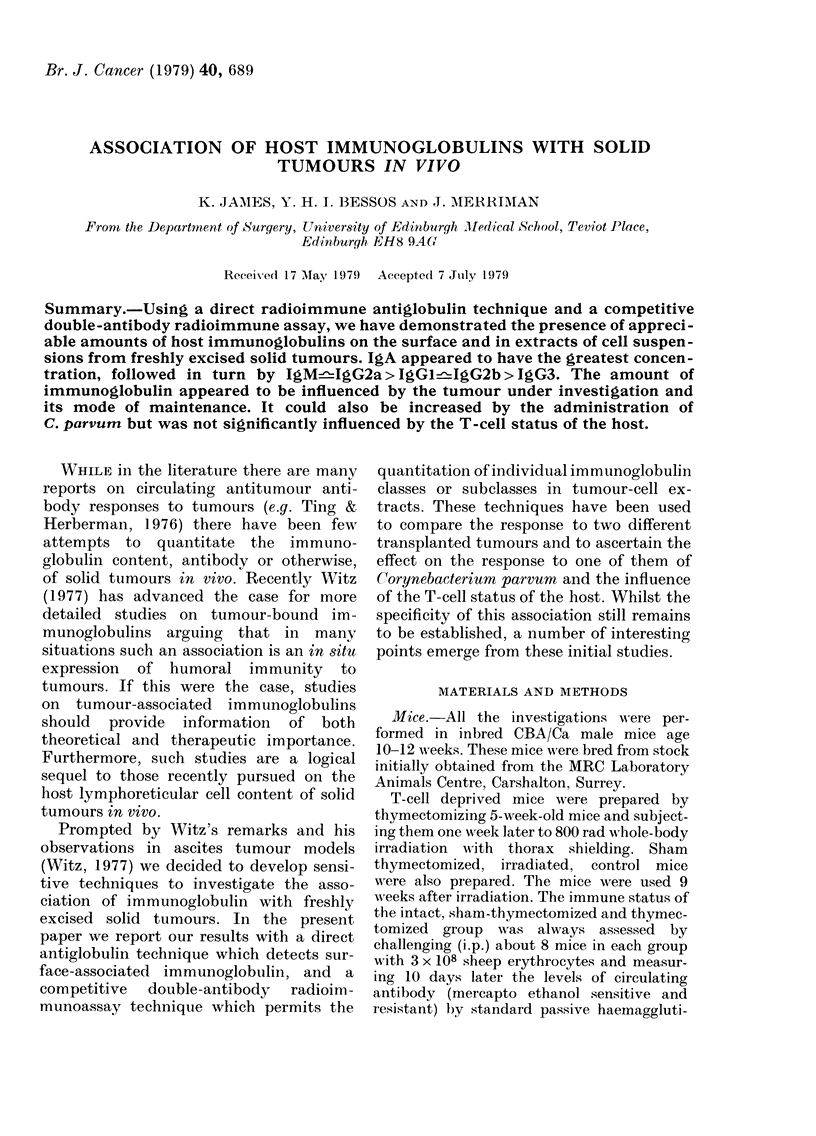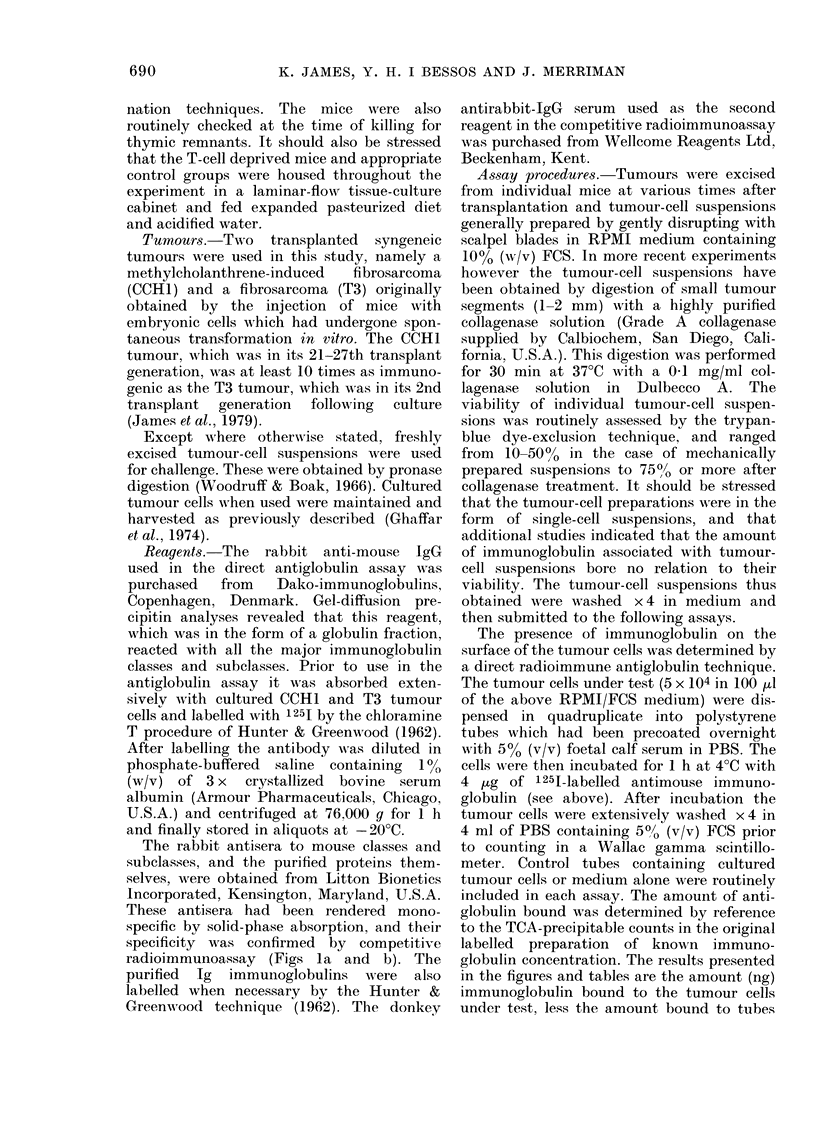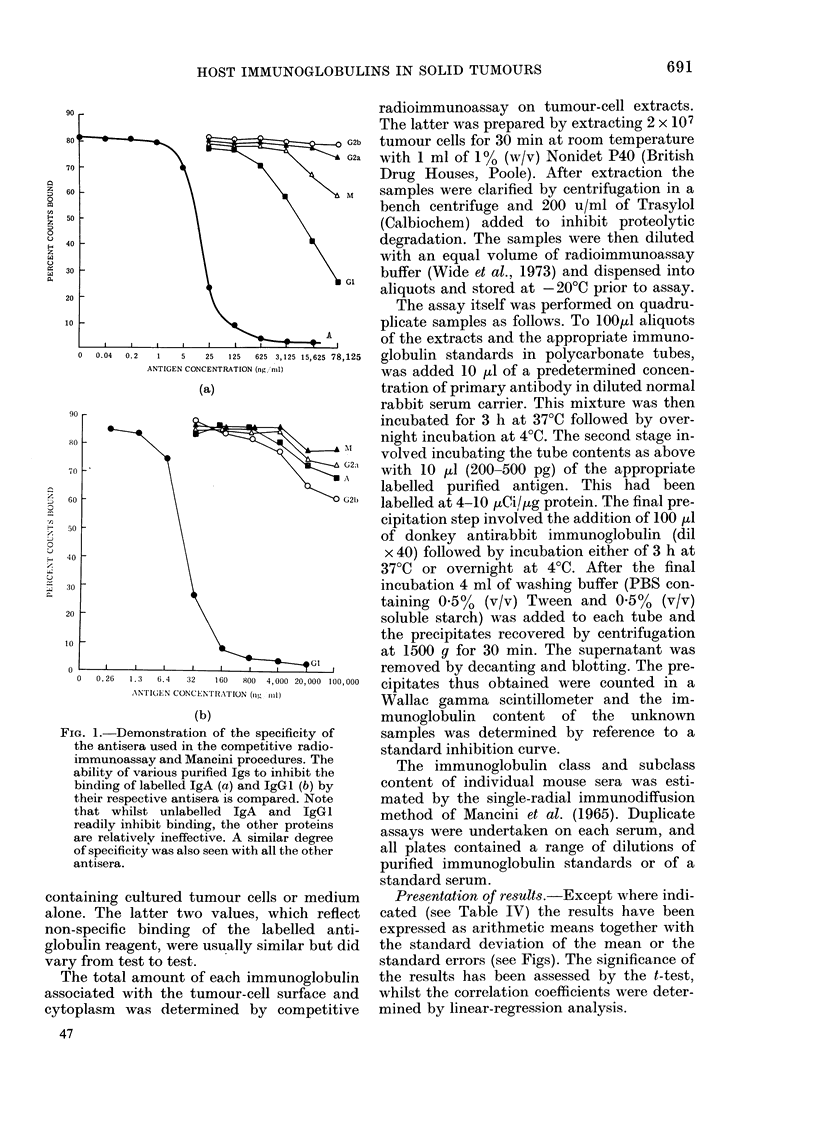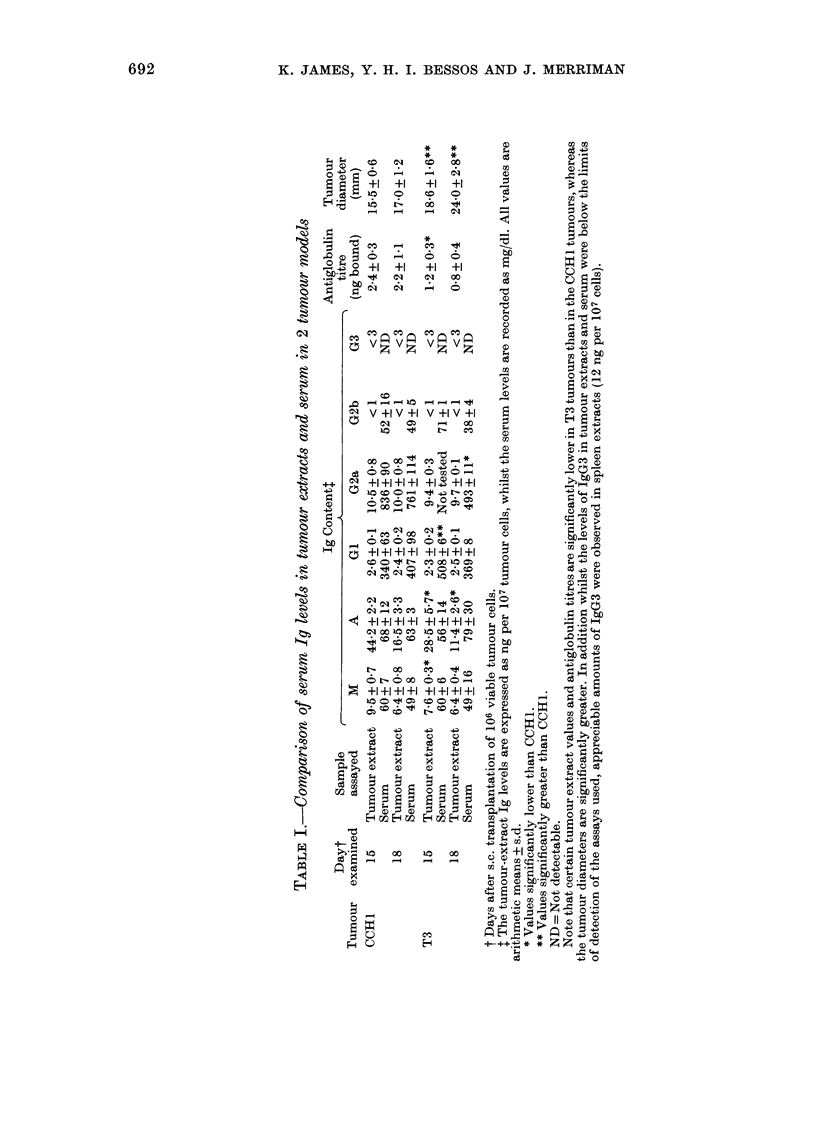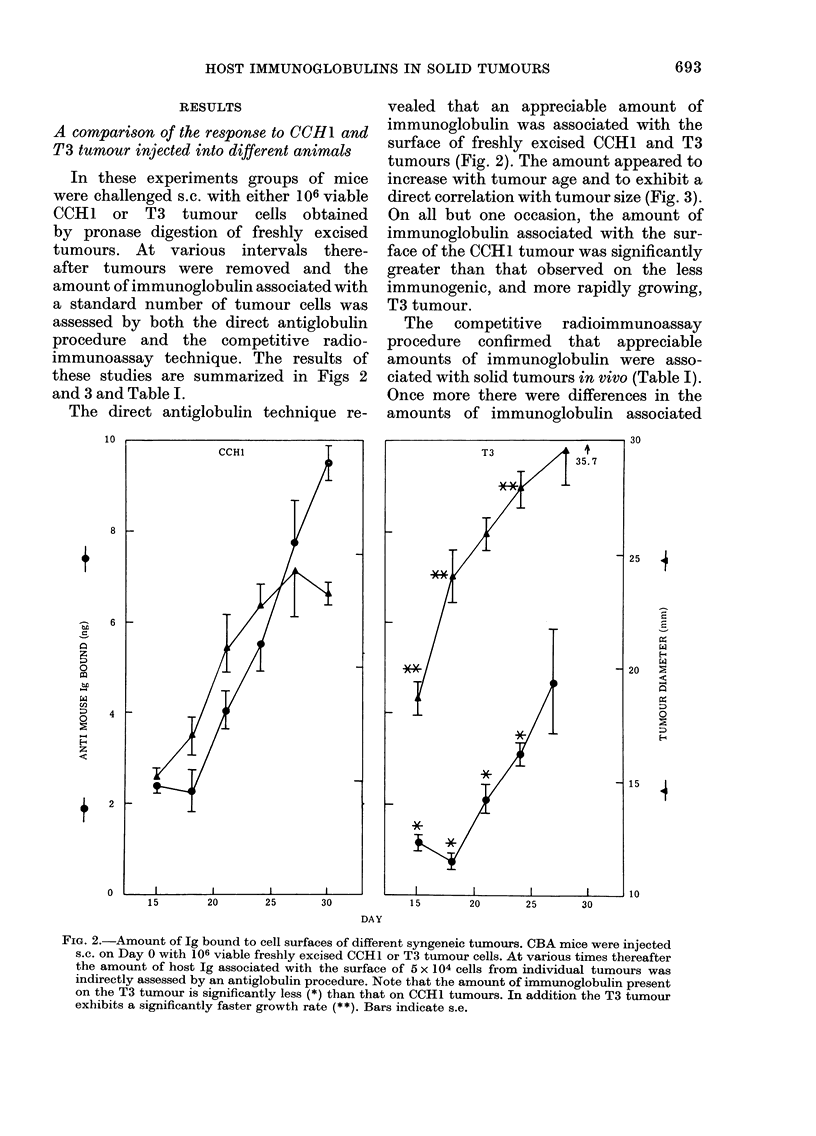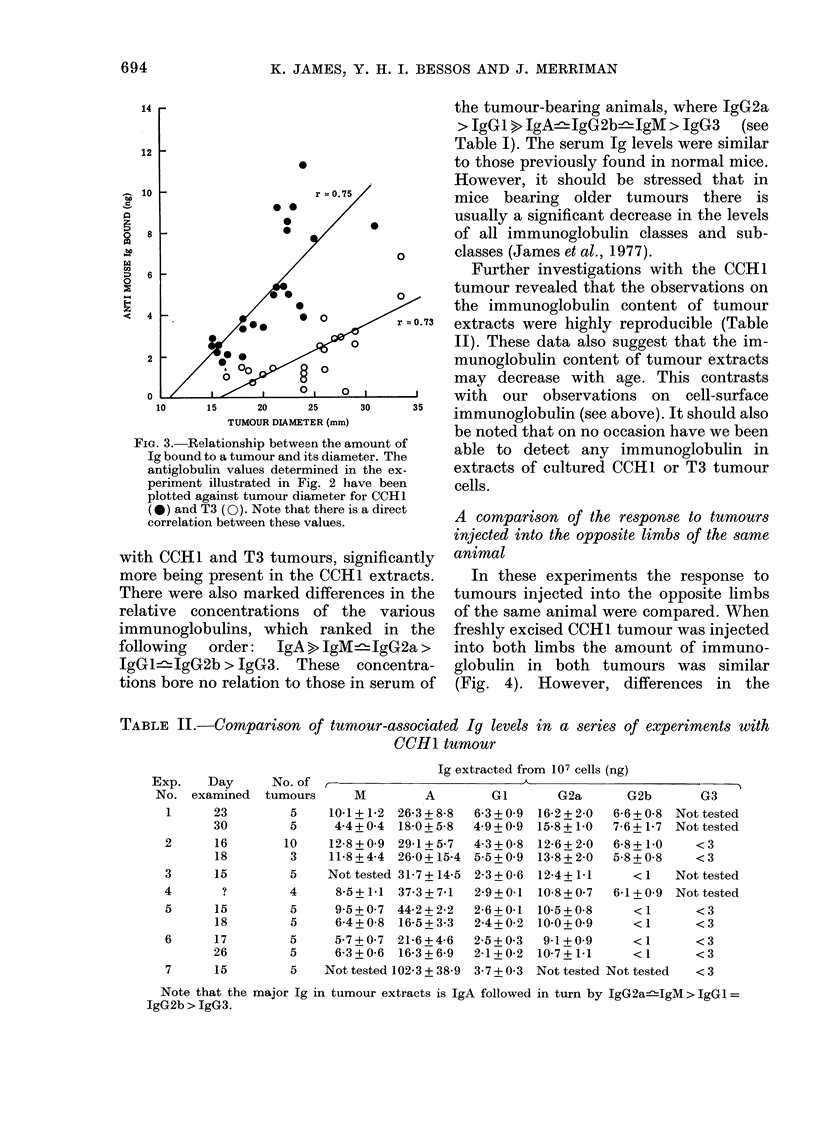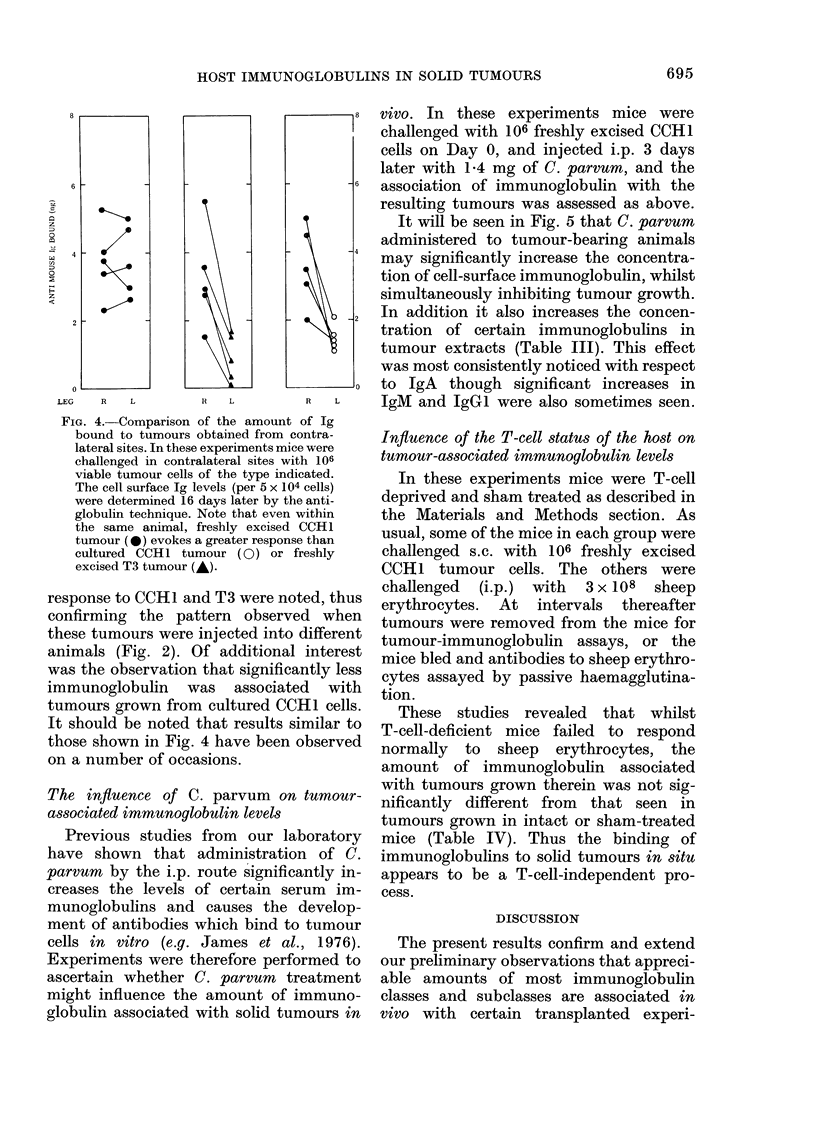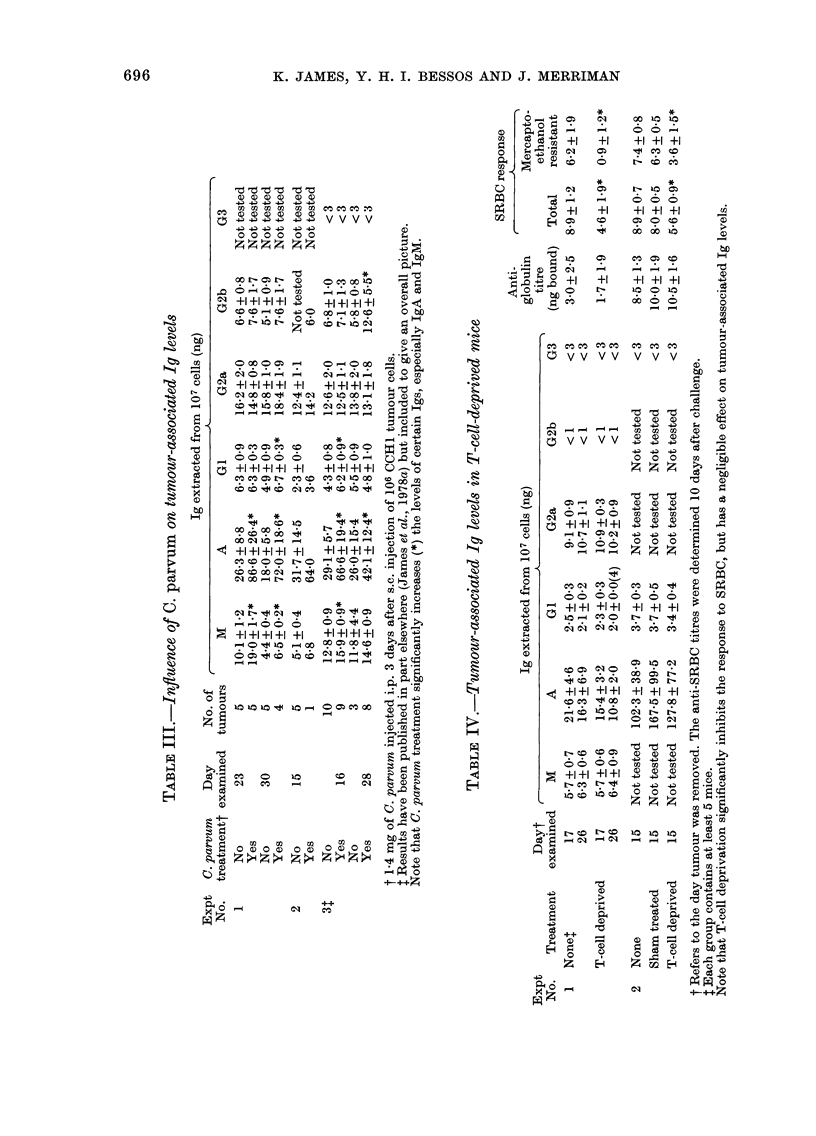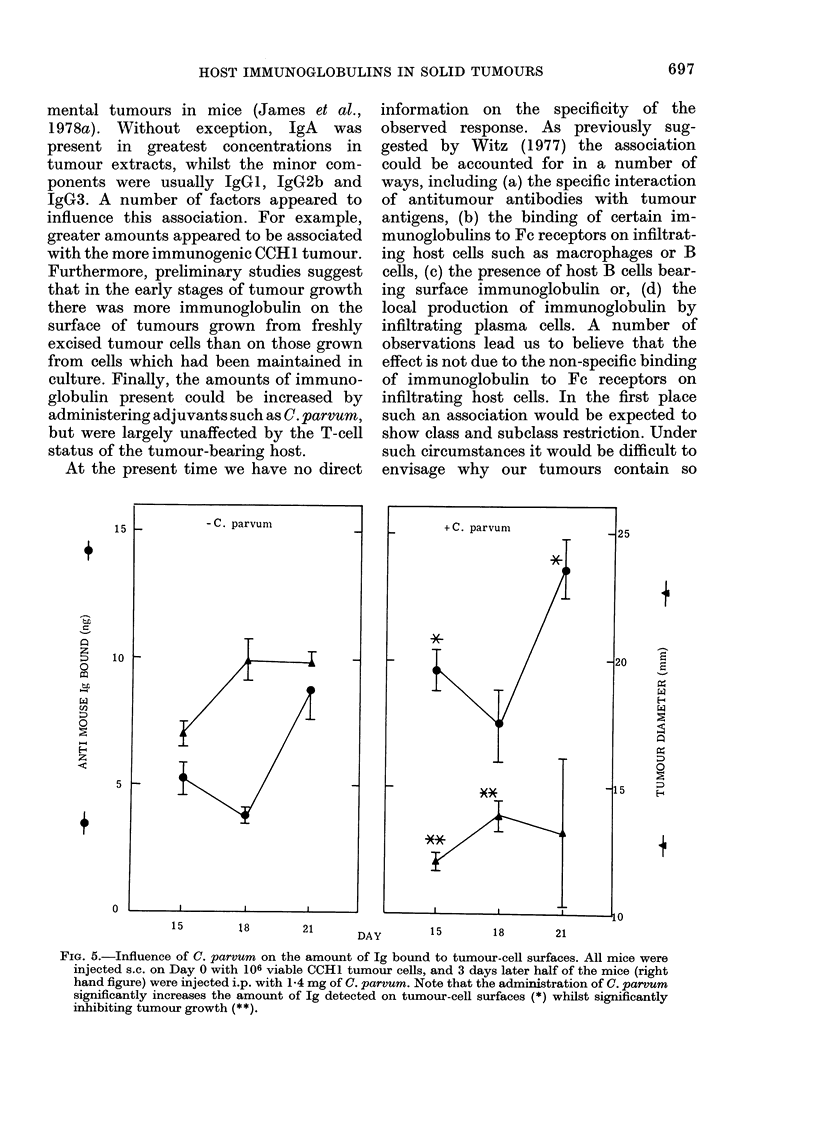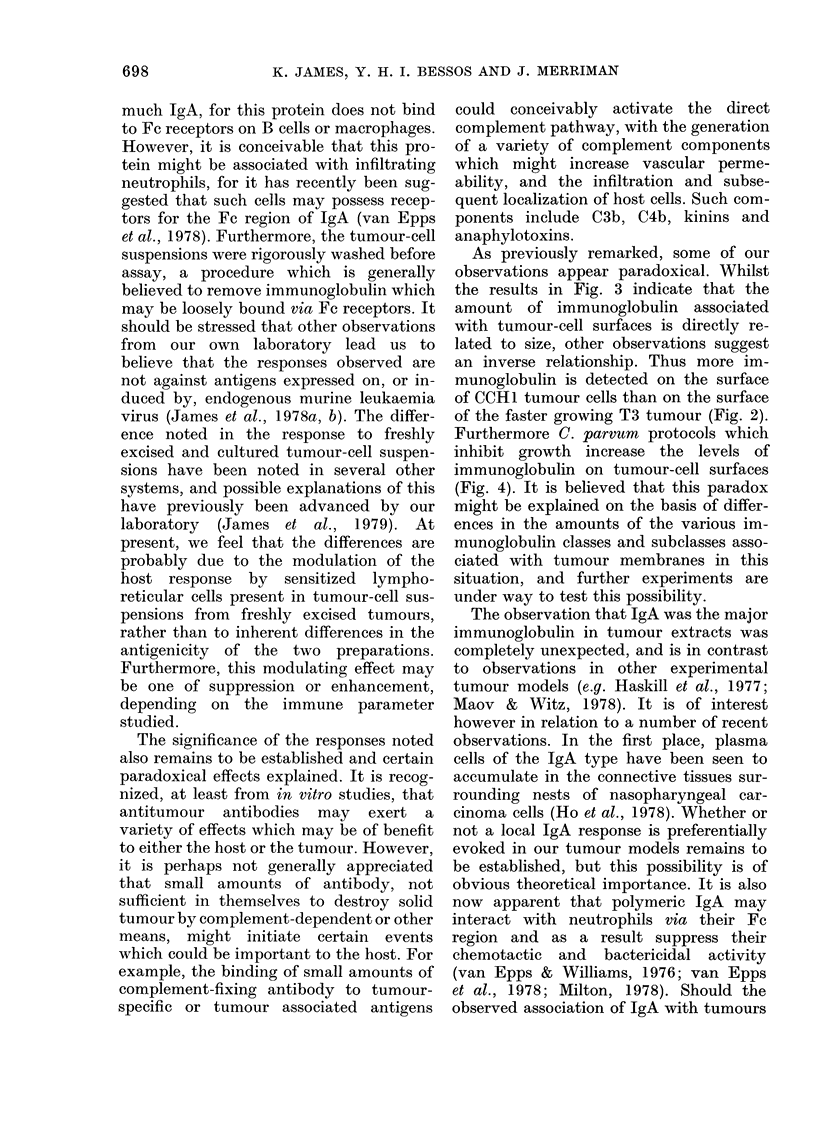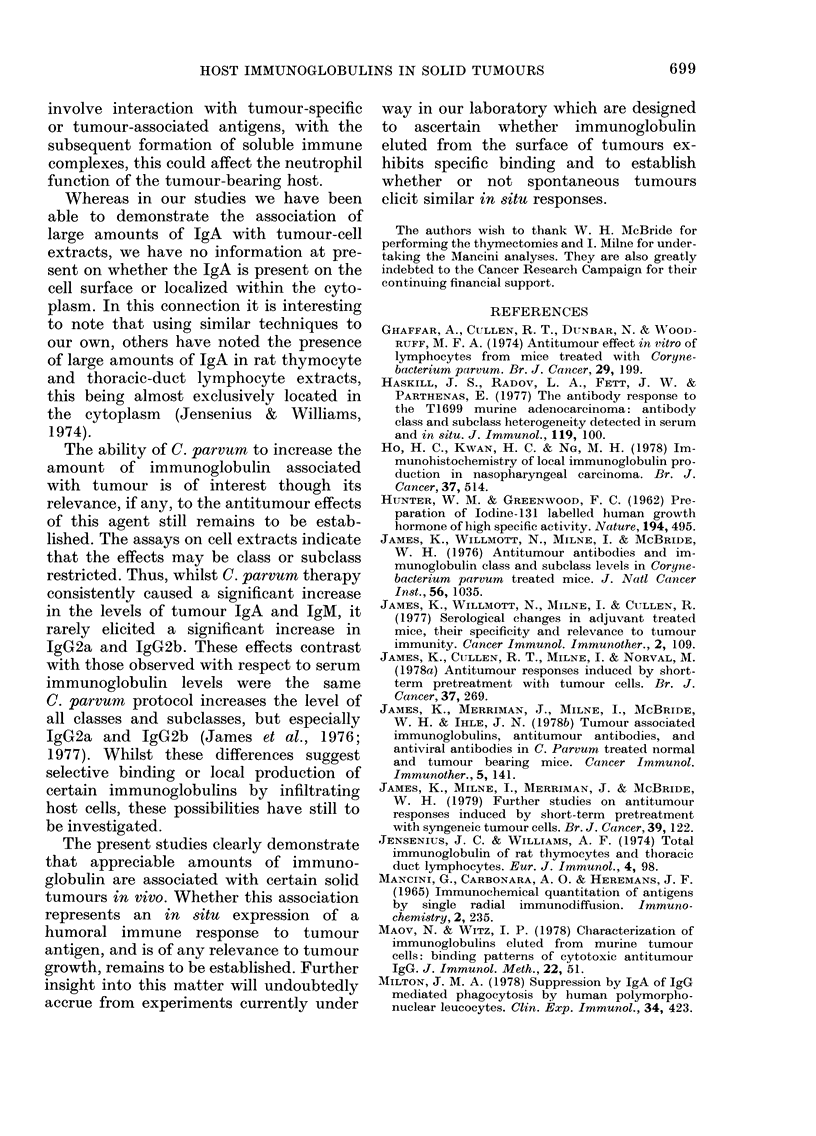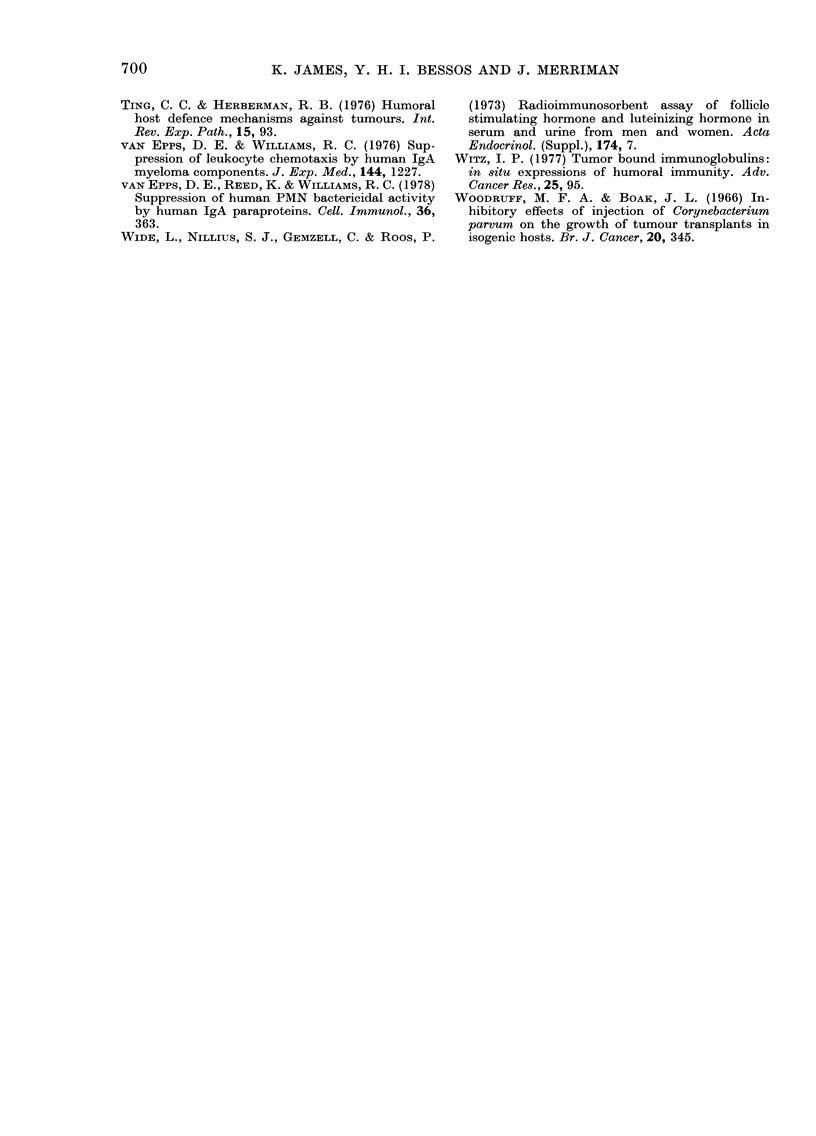# Association of host immunoglobulins with solid tumours in vivo.

**DOI:** 10.1038/bjc.1979.249

**Published:** 1979-11

**Authors:** K. James, Y. H. Bessos, J. Merriman

## Abstract

Using a direct radioimmune antiglobulin technique and a competitive double-antibody radioimmune assay, we have demonstrated the presence of appreciable amounts of host immunoglobulins on the surface and in extracts of cell suspensions from freshly excised solid tumours. IgA appeared to have the greatest concentrations from freshly excised solid tumours. IgA appeared to have the greatest concentration, followed in turn by IgM congruent to IgG2a greater than IgG1 congruent to IgG2b greater than IgG3. The amount of immunoglobulin appeared to be influenced by the tumour under investigation and its mode of maintenance. It could also be increased by the administration of C. parvum but was not significantly influenced by the T-cell status of the host.


*Br. J. Cancer* (1979) **40**, 689

### ASSOCIATION OF HOST IMMUNOGLOBULINS WITH SOLID TUMOURS *IN VIVO*

K. JAMES, Y. H. I. BESSOS AND J. MERRIMAN

*From the Department of Surgery, University of Edinburgh Medical School, Teviot Place, Edinburgh EH8 9AG*

Received 17 May 1979 Accepted 7 July 1979

**Summary.—Using a direct radioimmune antiglobulin technique and a competitive double-antibody radioimmune assay, we have demonstrated the presence of appreciable amounts of host immunoglobulins on the surface and in extracts of cell suspensions from freshly excised solid tumours. IgA appeared to have the greatest concentration, followed in turn by IgM≏IgG2a > IgG1≏IgG2b > IgG3. The amount of immunoglobulin appeared to be influenced by the tumour under investigation and its mode of maintenance. It could also be increased by the administration of *C. parvum* but was not significantly influenced by the T-cell status of the host.**

WHILE in the literature there are many reports on circulating antitumour antibody responses to tumours (*e.g.* Ting & Herberman, 1976) there have been few attempts to quantitate the immunoglobulin content, antibody or otherwise, of solid tumours *in vivo*. Recently Witz (1977) has advanced the case for more detailed studies on tumour-bound immunoglobulins arguing that in many situations such an association is an *in situ* expression of humoral immunity to tumours. If this were the case, studies on tumour-associated immunoglobulins should provide information of both theoretical and therapeutic importance. Furthermore, such studies are a logical sequel to those recently pursued on the host lymphoreticular cell content of solid tumours *in vivo*.

Prompted by Witz's remarks and his observations in ascites tumour models (Witz, 1977) we decided to develop sensitive techniques to investigate the association of immunoglobulin with freshly excised solid tumours. In the present paper we report our results with a direct antiglobulin technique which detects surface-associated immunoglobulin, and a competitive double-antibody radioimmunoassay technique which permits the quantitation of individual immunoglobulin classes or subclasses in tumour-cell extracts. These techniques have been used to compare the response to two different transplanted tumours and to ascertain the effect on the response to one of them of *Corynebacterium parvum* and the influence of the T-cell status of the host. Whilst the specificity of this association still remains to be established, a number of interesting points emerge from these initial studies.

### MATERIALS AND METHODS

*Mice*.—All the investigations were performed in inbred CBA/Ca male mice age 10–12 weeks. These mice were bred from stock initially obtained from the MRC Laboratory Animals Centre, Carshalton, Surrey.

T-cell deprived mice were prepared by thymectomizing 5-week-old mice and subjecting them one week later to 800 rad whole-body irradiation with thorax shielding. Sham thymectomized, irradiated, control mice were also prepared. The mice were used 9 weeks after irradiation. The immune status of the intact, sham-thymectomized and thymectomized group was always assessed by challenging (i.p.) about 8 mice in each group with $3 \times 10^8$ sheep erythrocytes and measuring 10 days later the levels of circulating antibody (mercapto ethanol sensitive and resistant) by standard passive haemagglutination techniques. The mice were also routinely checked at the time of killing for thymic remnants. It should also be stressed that the T-cell deprived mice and appropriate control groups were housed throughout the experiment in a laminar-flow tissue-culture cabinet and fed expanded pasteurized diet and acidified water.

*Tumours.*—Two transplanted syngeneic tumours were used in this study, namely a methylcholanthrene-induced fibrosarcoma (CCH1) and a fibrosarcoma (T3) originally obtained by the injection of mice with embryonic cells which had undergone spontaneous transformation *in vitro*. The CCH1 tumour, which was in its 21–27th transplant generation, was at least 10 times as immunogenic as the T3 tumour, which was in its 2nd transplant generation following culture (James *et al.*, 1979).

Except where otherwise stated, freshly excised tumour-cell suspensions were used for challenge. These were obtained by pronase digestion (Woodruff & Boak, 1966). Cultured tumour cells when used were maintained and harvested as previously described (Ghaffar *et al.*, 1974).

*Reagents.*—The rabbit anti-mouse IgG used in the direct antiglobulin assay was purchased from Dako-immunoglobulins, Copenhagen, Denmark. Gel-diffusion precipitin analyses revealed that this reagent, which was in the form of a globulin fraction, reacted with all the major immunoglobulin classes and subclasses. Prior to use in the antiglobulin assay it was absorbed extensively with cultured CCH1 and T3 tumour cells and labelled with $^{125}$I by the chloramine T procedure of Hunter & Greenwood (1962). After labelling the antibody was diluted in phosphate-buffered saline containing 1% (w/v) of 3× crystallized bovine serum albumin (Armour Pharmaceuticals, Chicago, U.S.A.) and centrifuged at 76,000 *g* for 1 h and finally stored in aliquots at −20°C.

The rabbit antisera to mouse classes and subclasses, and the purified proteins themselves, were obtained from Litton Bionetics Incorporated, Kensington, Maryland, U.S.A. These antisera had been rendered monospecific by solid-phase absorption, and their specificity was confirmed by competitive radioimmunoassay (Figs 1a and b). The purified Ig immunoglobulins were also labelled when necessary by the Hunter & Greenwood technique (1962). The donkey antirabbit-IgG serum used as the second reagent in the competitive radioimmunoassay was purchased from Wellcome Reagents Ltd, Beckenham, Kent.

*Assay procedures.*—Tumours were excised from individual mice at various times after transplantation and tumour-cell suspensions generally prepared by gently disrupting with scalpel blades in RPMI medium containing 10% (w/v) FCS. In more recent experiments however the tumour-cell suspensions have been obtained by digestion of small tumour segments (1–2 mm) with a highly purified collagenase solution (Grade A collagenase supplied by Calbiochem, San Diego, California, U.S.A.). This digestion was performed for 30 min at 37°C with a 0·1 mg/ml collagenase solution in Dulbecco A. The viability of individual tumour-cell suspensions was routinely assessed by the trypan-blue dye-exclusion technique, and ranged from 10–50% in the case of mechanically prepared suspensions to 75% or more after collagenase treatment. It should be stressed that the tumour-cell preparations were in the form of single-cell suspensions, and that additional studies indicated that the amount of immunoglobulin associated with tumour-cell suspensions bore no relation to their viability. The tumour-cell suspensions thus obtained were washed ×4 in medium and then submitted to the following assays.

The presence of immunoglobulin on the surface of the tumour cells was determined by a direct radioimmune antiglobulin technique. The tumour cells under test ($5 \times 10^4$ in 100 μl of the above RPMI/FCS medium) were dispensed in quadruplicate into polystyrene tubes which had been precoated overnight with 5% (v/v) foetal calf serum in PBS. The cells were then incubated for 1 h at 4°C with 4 μg of $^{125}$I-labelled antimouse immunoglobulin (see above). After incubation the tumour cells were extensively washed ×4 in 4 ml of PBS containing 5% (v/v) FCS prior to counting in a Wallac gamma scintillometer. Control tubes containing cultured tumour cells or medium alone were routinely included in each assay. The amount of antiglobulin bound was determined by reference to the TCA-precipitable counts in the original labelled preparation of known immunoglobulin concentration. The results presented in the figures and tables are the amount (ng) immunoglobulin bound to the tumour cells under test, less the amount bound to tubes

Fig. 1.—Demonstration of the specificity of the antisera used in the competitive radioimmunoassay and Mancini procedures. The ability of various purified Igs to inhibit the binding of labelled IgA (*a*) and IgG1 (*b*) by their respective antisera is compared. Note that whilst unlabelled IgA and IgG1 readily inhibit binding, the other proteins are relatively ineffective. A similar degree of specificity was also seen with all the other antisera.

containing cultured tumour cells or medium alone. The latter two values, which reflect non-specific binding of the labelled antiglobulin reagent, were usually similar but did vary from test to test.

The total amount of each immunoglobulin associated with the tumour-cell surface and cytoplasm was determined by competitive radioimmunoassay on tumour-cell extracts. The latter was prepared by extracting $2 \times 10^7$ tumour cells for 30 min at room temperature with 1 ml of 1% (w/v) Nonidet P40 (British Drug Houses, Poole). After extraction the samples were clarified by centrifugation in a bench centrifuge and 200 u/ml of Trasylol (Calbiochem) added to inhibit proteolytic degradation. The samples were then diluted with an equal volume of radioimmunoassay buffer (Wide *et al.*, 1973) and dispensed into aliquots and stored at −20°C prior to assay.

The assay itself was performed on quadruplicate samples as follows. To 100 μl aliquots of the extracts and the appropriate immunoglobulin standards in polycarbonate tubes, was added 10 μl of a predetermined concentration of primary antibody in diluted normal rabbit serum carrier. This mixture was then incubated for 3 h at 37°C followed by overnight incubation at 4°C. The second stage involved incubating the tube contents as above with 10 μl (200–500 pg) of the appropriate labelled purified antigen. This had been labelled at 4–10 μCi/μg protein. The final precipitation step involved the addition of 100 μl of donkey antirabbit immunoglobulin (dil ×40) followed by incubation either of 3 h at 37°C or overnight at 4°C. After the final incubation 4 ml of washing buffer (PBS containing 0·5% (v/v) Tween and 0·5% (v/v) soluble starch) was added to each tube and the precipitates recovered by centrifugation at 1500 *g* for 30 min. The supernatant was removed by decanting and blotting. The precipitates thus obtained were counted in a Wallac gamma scintillometer and the immunoglobulin content of the unknown samples was determined by reference to a standard inhibition curve.

The immunoglobulin class and subclass content of individual mouse sera was estimated by the single-radial immunodiffusion method of Mancini *et al.* (1965). Duplicate assays were undertaken on each serum, and all plates contained a range of dilutions of purified immunoglobulin standards or of a standard serum.

*Presentation of results.*—Except where indicated (see Table IV) the results have been expressed as arithmetic means together with the standard deviation of the mean or the standard errors (see Figs). The significance of the results has been assessed by the *t*-test, whilst the correlation coefficients were determined by linear-regression analysis.

### RESULTS

### *A comparison of the response to CCH1 and T3 tumour injected into different animals*

In these experiments groups of mice were challenged s.c. with either $10^6$ viable CCH1 or T3 tumour cells obtained by pronase digestion of freshly excised tumours. At various intervals thereafter tumours were removed and the amount of immunoglobulin associated with a standard number of tumour cells was assessed by both the direct antiglobulin procedure and the competitive radioimmunoassay technique. The results of these studies are summarized in Figs 2 and 3 and Table I.

The direct antiglobulin technique revealed that an appreciable amount of immunoglobulin was associated with the surface of freshly excised CCH1 and T3 tumours (Fig. 2). The amount appeared to increase with tumour age and to exhibit a direct correlation with tumour size (Fig. 3). On all but one occasion, the amount of immunoglobulin associated with the surface of the CCH1 tumour was significantly greater than that observed on the less immunogenic, and more rapidly growing, T3 tumour.

The competitive radioimmunoassay procedure confirmed that appreciable amounts of immunoglobulin were associated with solid tumours *in vivo* (Table I). Once more there were differences in the amounts of immunoglobulin associated

FIG. 2.—Amount of Ig bound to cell surfaces of different syngeneic tumours. CBA mice were injected s.c. on Day 0 with $10^6$ viable freshly excised CCH1 or T3 tumour cells. At various times thereafter the amount of host Ig associated with the surface of $5 \times 10^4$ cells from individual tumours was indirectly assessed by an antiglobulin procedure. Note that the amount of immunoglobulin present on the T3 tumour is significantly less (*) than that on CCH1 tumours. In addition the T3 tumour exhibits a significantly faster growth rate (**). Bars indicate s.e.

FIG. 3.—Relationship between the amount of Ig bound to a tumour and its diameter. The antiglobulin values determined in the experiment illustrated in Fig. 2 have been plotted against tumour diameter for CCH1 (●) and T3 (○). Note that there is a direct correlation between these values.

with CCH1 and T3 tumours, significantly more being present in the CCH1 extracts. There were also marked differences in the relative concentrations of the various immunoglobulins, which ranked in the following order: IgA ≫ IgM≃IgG2a > IgG1≃IgG2b > IgG3. These concentrations bore no relation to those in serum of the tumour-bearing animals, where IgG2a > IgG1 ≫ IgA≃IgG2b≃IgM > IgG3 (see Table I). The serum Ig levels were similar to those previously found in normal mice. However, it should be stressed that in mice bearing older tumours there is usually a significant decrease in the levels of all immunoglobulin classes and subclasses (James *et al.*, 1977).

Further investigations with the CCH1 tumour revealed that the observations on the immunoglobulin content of tumour extracts were highly reproducible (Table II). These data also suggest that the immunoglobulin content of tumour extracts may decrease with age. This contrasts with our observations on cell-surface immunoglobulin (see above). It should also be noted that on no occasion have we been able to detect any immunoglobulin in extracts of cultured CCH1 or T3 tumour cells.

*A comparison of the response to tumours injected into the opposite limbs of the same animal*

In these experiments the response to tumours injected into the opposite limbs of the same animal were compared. When freshly excised CCH1 tumour was injected into both limbs the amount of immunoglobulin in both tumours was similar (Fig. 4). However, differences in the

TABLE II.—*Comparison of tumour-associated Ig levels in a series of experiments with CCH1 tumour*

| Exp. No. | Day examined | No. of tumours | Ig extracted from $10^7$ cells (ng) | | | | | |
|---|---|---|---|---|---|---|---|---|
| | | | M | A | G1 | G2a | G2b | G3 |
| 1 | 23 | 5 | 10·1 ± 1·2 | 26·3 ± 8·8 | 6·3 ± 0·9 | 16·2 ± 2·0 | 6·6 ± 0·8 | Not tested |
| | 30 | 5 | 4·4 ± 0·4 | 18·0 ± 5·8 | 4·9 ± 0·9 | 15·8 ± 1·0 | 7·6 ± 1·7 | Not tested |
| 2 | 16 | 10 | 12·8 ± 0·9 | 29·1 ± 5·7 | 4·3 ± 0·8 | 12·6 ± 2·0 | 6·8 ± 1·0 | < 3 |
| | 18 | 3 | 11·8 ± 4·4 | 26·0 ± 15·4 | 5·5 ± 0·9 | 13·8 ± 2·0 | 5·8 ± 0·8 | < 3 |
| 3 | 15 | 5 | Not tested | 31·7 ± 14·5 | 2·3 ± 0·6 | 12·4 ± 1·1 | < 1 | Not tested |
| 4 | ? | 4 | 8·5 ± 1·1 | 37·3 ± 7·1 | 2·9 ± 0·1 | 10·8 ± 0·7 | 6·1 ± 0·9 | Not tested |
| 5 | 15 | 5 | 9·5 ± 0·7 | 44·2 ± 2·2 | 2·6 ± 0·1 | 10·5 ± 0·8 | < 1 | < 3 |
| | 18 | 5 | 6·4 ± 0·8 | 16·5 ± 3·3 | 2·4 ± 0·2 | 10·0 ± 0·9 | < 1 | < 3 |
| 6 | 17 | 5 | 5·7 ± 0·7 | 21·6 ± 4·6 | 2·5 ± 0·3 | 9·1 ± 0·9 | < 1 | < 3 |
| | 26 | 5 | 6·3 ± 0·6 | 16·3 ± 6·9 | 2·1 ± 0·2 | 10·7 ± 1·1 | < 1 | < 3 |
| 7 | 15 | 5 | Not tested | 102·3 ± 38·9 | 3·7 ± 0·3 | Not tested | Not tested | < 3 |

Note that the major Ig in tumour extracts is IgA followed in turn by IgG2a≃IgM > IgG1 = IgG2b > IgG3.

FIG. 4.—Comparison of the amount of Ig bound to tumours obtained from contralateral sites. In these experiments mice were challenged in contralateral sites with $10^6$ viable tumour cells of the type indicated. The cell surface Ig levels (per $5 \times 10^4$ cells) were determined 16 days later by the antiglobulin technique. Note that even within the same animal, freshly excised CCH1 tumour (●) evokes a greater response than cultured CCH1 tumour (○) or freshly excised T3 tumour (▲).

response to CCH1 and T3 were noted, thus confirming the pattern observed when these tumours were injected into different animals (Fig. 2). Of additional interest was the observation that significantly less immunoglobulin was associated with tumours grown from cultured CCH1 cells. It should be noted that results similar to those shown in Fig. 4 have been observed on a number of occasions.

### *The influence of* C. parvum *on tumour-associated immunoglobulin levels*

Previous studies from our laboratory have shown that administration of *C. parvum* by the i.p. route significantly increases the levels of certain serum immunoglobulins and causes the development of antibodies which bind to tumour cells *in vitro* (*e.g.* James *et al.*, 1976). Experiments were therefore performed to ascertain whether *C. parvum* treatment might influence the amount of immunoglobulin associated with solid tumours *in vivo*. In these experiments mice were challenged with $10^6$ freshly excised CCH1 cells on Day 0, and injected i.p. 3 days later with 1·4 mg of *C. parvum*, and the association of immunoglobulin with the resulting tumours was assessed as above.

It will be seen in Fig. 5 that *C. parvum* administered to tumour-bearing animals may significantly increase the concentration of cell-surface immunoglobulin, whilst simultaneously inhibiting tumour growth. In addition it also increases the concentration of certain immunoglobulins in tumour extracts (Table III). This effect was most consistently noticed with respect to IgA though significant increases in IgM and IgG1 were also sometimes seen.

### *Influence of the T-cell status of the host on tumour-associated immunoglobulin levels*

In these experiments mice were T-cell deprived and sham treated as described in the Materials and Methods section. As usual, some of the mice in each group were challenged s.c. with $10^6$ freshly excised CCH1 tumour cells. The others were challenged (i.p.) with $3 \times 10^8$ sheep erythrocytes. At intervals thereafter tumours were removed from the mice for tumour-immunoglobulin assays, or the mice bled and antibodies to sheep erythrocytes assayed by passive haemagglutination.

These studies revealed that whilst T-cell-deficient mice failed to respond normally to sheep erythrocytes, the amount of immunoglobulin associated with tumours grown therein was not significantly different from that seen in tumours grown in intact or sham-treated mice (Table IV). Thus the binding of immunoglobulins to solid tumours *in situ* appears to be a T-cell-independent process.

### DISCUSSION

The present results confirm and extend our preliminary observations that appreciable amounts of most immunoglobulin classes and subclasses are associated *in vivo* with certain transplanted experimental tumours in mice (James *et al.*, 1978*a*). Without exception, IgA was present in greatest concentrations in tumour extracts, whilst the minor components were usually IgG1, IgG2b and IgG3. A number of factors appeared to influence this association. For example, greater amounts appeared to be associated with the more immunogenic CCH1 tumour. Furthermore, preliminary studies suggest that in the early stages of tumour growth there was more immunoglobulin on the surface of tumours grown from freshly excised tumour cells than on those grown from cells which had been maintained in culture. Finally, the amounts of immunoglobulin present could be increased by administering adjuvants such as *C. parvum*, but were largely unaffected by the T-cell status of the tumour-bearing host.

At the present time we have no direct information on the specificity of the observed response. As previously suggested by Witz (1977) the association could be accounted for in a number of ways, including (a) the specific interaction of antitumour antibodies with tumour antigens, (b) the binding of certain immunoglobulins to Fc receptors on infiltrating host cells such as macrophages or B cells, (c) the presence of host B cells bearing surface immunoglobulin or, (d) the local production of immunoglobulin by infiltrating plasma cells. A number of observations lead us to believe that the effect is not due to the non-specific binding of immunoglobulin to Fc receptors on infiltrating host cells. In the first place such an association would be expected to show class and subclass restriction. Under such circumstances it would be difficult to envisage why our tumours contain so

FIG. 5.—Influence of *C. parvum* on the amount of Ig bound to tumour-cell surfaces. All mice were injected s.c. on Day 0 with $10^6$ viable CCH1 tumour cells, and 3 days later half of the mice (right hand figure) were injected i.p. with 1·4 mg of *C. parvum*. Note that the administration of *C. parvum* significantly increases the amount of Ig detected on tumour-cell surfaces (*) whilst significantly inhibiting tumour growth (**).

much IgA, for this protein does not bind to Fc receptors on B cells or macrophages. However, it is conceivable that this protein might be associated with infiltrating neutrophils, for it has recently been suggested that such cells may possess receptors for the Fc region of IgA (van Epps *et al.*, 1978). Furthermore, the tumour-cell suspensions were rigorously washed before assay, a procedure which is generally believed to remove immunoglobulin which may be loosely bound *via* Fc receptors. It should be stressed that other observations from our own laboratory lead us to believe that the responses observed are not against antigens expressed on, or induced by, endogenous murine leukaemia virus (James *et al.*, 1978*a*, *b*). The difference noted in the response to freshly excised and cultured tumour-cell suspensions have been noted in several other systems, and possible explanations of this have previously been advanced by our laboratory (James *et al.*, 1979). At present, we feel that the differences are probably due to the modulation of the host response by sensitized lymphoreticular cells present in tumour-cell suspensions from freshly excised tumours, rather than to inherent differences in the antigenicity of the two preparations. Furthermore, this modulating effect may be one of suppression or enhancement, depending on the immune parameter studied.

The significance of the responses noted also remains to be established and certain paradoxical effects explained. It is recognized, at least from *in vitro* studies, that antitumour antibodies may exert a variety of effects which may be of benefit to either the host or the tumour. However, it is perhaps not generally appreciated that small amounts of antibody, not sufficient in themselves to destroy solid tumour by complement-dependent or other means, might initiate certain events which could be important to the host. For example, the binding of small amounts of complement-fixing antibody to tumour-specific or tumour associated antigens could conceivably activate the direct complement pathway, with the generation of a variety of complement components which might increase vascular permeability, and the infiltration and subsequent localization of host cells. Such components include C3b, C4b, kinins and anaphylotoxins.

As previously remarked, some of our observations appear paradoxical. Whilst the results in Fig. 3 indicate that the amount of immunoglobulin associated with tumour-cell surfaces is directly related to size, other observations suggest an inverse relationship. Thus more immunoglobulin is detected on the surface of CCH1 tumour cells than on the surface of the faster growing T3 tumour (Fig. 2). Furthermore *C. parvum* protocols which inhibit growth increase the levels of immunoglobulin on tumour-cell surfaces (Fig. 4). It is believed that this paradox might be explained on the basis of differences in the amounts of the various immunoglobulin classes and subclasses associated with tumour membranes in this situation, and further experiments are under way to test this possibility.

The observation that IgA was the major immunoglobulin in tumour extracts was completely unexpected, and is in contrast to observations in other experimental tumour models (*e.g.* Haskill *et al.*, 1977; Maov & Witz, 1978). It is of interest however in relation to a number of recent observations. In the first place, plasma cells of the IgA type have been seen to accumulate in the connective tissues surrounding nests of nasopharyngeal carcinoma cells (Ho *et al.*, 1978). Whether or not a local IgA response is preferentially evoked in our tumour models remains to be established, but this possibility is of obvious theoretical importance. It is also now apparent that polymeric IgA may interact with neutrophils *via* their Fc region and as a result suppress their chemotactic and bactericidal activity (van Epps & Williams, 1976; van Epps *et al.*, 1978; Milton, 1978). Should the observed association of IgA with tumours involve interaction with tumour-specific or tumour-associated antigens, with the subsequent formation of soluble immune complexes, this could affect the neutrophil function of the tumour-bearing host.

Whereas in our studies we have been able to demonstrate the association of large amounts of IgA with tumour-cell extracts, we have no information at present on whether the IgA is present on the cell surface or localized within the cytoplasm. In this connection it is interesting to note that using similar techniques to our own, others have noted the presence of large amounts of IgA in rat thymocyte and thoracic-duct lymphocyte extracts, this being almost exclusively located in the cytoplasm (Jensenius & Williams, 1974).

The ability of *C. parvum* to increase the amount of immunoglobulin associated with tumour is of interest though its relevance, if any, to the antitumour effects of this agent still remains to be established. The assays on cell extracts indicate that the effects may be class or subclass restricted. Thus, whilst *C. parvum* therapy consistently caused a significant increase in the levels of tumour IgA and IgM, it rarely elicited a significant increase in IgG2a and IgG2b. These effects contrast with those observed with respect to serum immunoglobulin levels were the same *C. parvum* protocol increases the level of all classes and subclasses, but especially IgG2a and IgG2b (James *et al.*, 1976; 1977). Whilst these differences suggest selective binding or local production of certain immunoglobulins by infiltrating host cells, these possibilities have still to be investigated.

The present studies clearly demonstrate that appreciable amounts of immunoglobulin are associated with certain solid tumours *in vivo*. Whether this association represents an *in situ* expression of a humoral immune response to tumour antigen, and is of any relevance to tumour growth, remains to be established. Further insight into this matter will undoubtedly accrue from experiments currently under way in our laboratory which are designed to ascertain whether immunoglobulin eluted from the surface of tumours exhibits specific binding and to establish whether or not spontaneous tumours elicit similar *in situ* responses.

The authors wish to thank W. H. McBride for performing the thymectomies and I. Milne for undertaking the Mancini analyses. They are also greatly indebted to the Cancer Research Campaign for their continuing financial support.

### REFERENCES

Ghaffar, A., Cullen, R. T., Dunbar, N. & Woodruff, M. F. A. (1974) Antitumour effect *in vitro* of lymphocytes from mice treated with *Corynebacterium parvum*. *Br. J. Cancer*, **29**, 199.

Haskill, J. S., Radov, L. A., Fett, J. W. & Parthenas, E. (1977) The antibody response to the T1699 murine adenocarcinoma: antibody class and subclass heterogeneity detected in serum and *in situ*. *J. Immunol.*, **119**, 100.

Ho, H. C., Kwan, H. C. & Ng, M. H. (1978) Immunohistochemistry of local immunoglobulin production in nasopharyngeal carcinoma. *Br. J. Cancer*, **37**, 514.

Hunter, W. M. & Greenwood, F. C. (1962) Preparation of Iodine-131 labelled human growth hormone of high specific activity. *Nature*, **194**, 495.

James, K., Willmott, N., Milne, I. & McBride, W. H. (1976) Antitumour antibodies and immunoglobulin class and subclass levels in *Corynebacterium parvum* treated mice. *J. Natl Cancer Inst.*, **56**, 1035.

James, K., Willmott, N., Milne, I. & Cullen, R. (1977) Serological changes in adjuvant treated mice, their specificity and relevance to tumour immunity. *Cancer Immunol. Immunother.*, **2**, 109.

James, K., Cullen, R. T., Milne, I. & Norval, M. (1978*a*) Antitumour responses induced by short-term pretreatment with tumour cells. *Br. J. Cancer*, **37**, 269.

James, K., Merriman, J., Milne, I., McBride, W. H. & Ihle, J. N. (1978*b*) Tumour associated immunoglobulins, antitumour antibodies, and antiviral antibodies in *C. Parvum* treated normal and tumour bearing mice. *Cancer Immunol. Immunother.*, **5**, 141.

James, K., Milne, I., Merriman, J. & McBride, W. H. (1979) Further studies on antitumour responses induced by short-term pretreatment with syngeneic tumour cells. *Br. J. Cancer*, **39**, 122.

Jensenius, J. C. & Williams, A. F. (1974) Total immunoglobulin of rat thymocytes and thoracic duct lymphocytes. *Eur. J. Immunol.*, **4**, 98.

Mancini, G., Carbonara, A. O. & Heremans, J. F. (1965) Immunochemical quantitation of antigens by single radial immunodiffusion. *Immunochemistry*, **2**, 235.

Maov, N. & Witz, I. P. (1978) Characterization of immunoglobulins eluted from murine tumour cells: binding patterns of cytotoxic antitumour IgG. *J. Immunol. Meth.*, **22**, 51.

Milton, J. M. A. (1978) Suppression by IgA of IgG mediated phagocytosis by human polymorphonuclear leucocytes. *Clin. Exp. Immunol.*, **34**, 423.

TING, C. C. & HERBERMAN, R. B. (1976) Humoral host defence mechanisms against tumours. *Int. Rev. Exp. Path.*, **15**, 93.

VAN EPPS, D. E. & WILLIAMS, R. C. (1976) Suppression of leukocyte chemotaxis by human IgA myeloma components. *J. Exp. Med.*, **144**, 1227.

VAN EPPS, D. E., REED, K. & WILLIAMS, R. C. (1978) Suppression of human PMN bactericidal activity by human IgA paraproteins. *Cell. Immunol.*, **36**, 363.

WIDE, L., NILLIUS, S. J., GEMZELL, C. & ROOS, P. (1973) Radioimmunosorbent assay of follicle stimulating hormone and luteinizing hormone in serum and urine from men and women. *Acta Endocrinol.* (Suppl.), **174**, 7.

WITZ, I. P. (1977) Tumor bound immunoglobulins: *in situ* expressions of humoral immunity. *Adv. Cancer Res.*, **25**, 95.

WOODRUFF, M. F. A. & BOAK, J. L. (1966) Inhibitory effects of injection of *Corynebacterium parvum* on the growth of tumour transplants in isogenic hosts. *Br. J. Cancer*, **20**, 345.